# Copper Availability Modulates Early Membrane-Associated Events in Polyethyleneimine Antifungal Activity in *Aspergillus nidulans*

**DOI:** 10.3390/jof12100746

**Published:** 2026-10-04

**Authors:** Spiros Gerostathis, Vicky Sophianopoulou

**Affiliations:** Microbial Molecular Genetics Laboratory, Institute of Biosciences and Applications (IBA), National Centre for Scientific Research “Demokritos”, 15341 Agia Paraskevi, Greece

**Keywords:** antifungal activity, polyethyleneimine (PEI), *Aspergillus nidulans*, copper homeostasis, plasma membrane

## Abstract

In healthy humans, a very low percentage of fungal species are capable of causing severe infections. On the contrary, in individuals with weak or compromised immune system, opportunistic fungal infections result in up to 3.8 million deaths annually. Drug resistance is the major problem for all of the currently available antifungals. Polyethyleneimine (PEI), a cationic polymer, exhibits potent antifungal activity against *Aspergillus nidulans*, yet the influence of copper ions on its mechanism of activity remains unclear. Here, we investigated how free exogenous Cu^2+^, influences PEI antifungal activity and the membrane-associated events underlying this effect. We demonstrate that (i) exogenous excess of homeostatic Cu^2+^ concentrations protects *A. nidulans* from PEI toxicity in a concentration-dependent and developmentally regulated manner; (ii) Cu^2+^ is effective only when present during the earliest stages of PEI action, identifying the plasma membrane as the primary site of Cu^2+^ protection; (iii) PEI, like Amphotericin B, induces translocation of ergosterol from the plasma membrane to intracellular compartments while additionally producing a distinct polarized enrichment of ergosterol at the hyphal tip; and (iv) Cu^2+^-mediated protection is independent of eisosome assembly. Together, these findings provide new mechanistic insights into the antifungal activity of PEI and the role of Cu^+2^ on PEI-induced cellular damage.

## 1. Introduction

Fungal infections represent a significant and growing cause of mortality worldwide, particularly among immunocompromised individuals. Globally, invasive fungal infections are estimated to cause approximately 3.8 million deaths annually, with Invasive Aspergillosis (IA) alone accounting for approximately 250,000 cases per year [1,2]. *Aspergillus fumigatus* is the primary causative agent of IA, although closely related species including *Aspergillus nidulans*, *Aspergillus terreus* and *Aspergillus flavus* are also clinically significant [3]. In addition to its role as an opportunistic pathogen, *A. nidulans* is a genetically tractable model filamentous fungus that has contributed extensively to our understanding of Aspergilli cell biology, membrane dynamics and stress responses, making it particularly well suited for mechanistic studies [3,4,5]. Despite advances in diagnosis and management, IA-associated mortality remains unacceptably high, approaching 100% in cases of disseminated infection [6,7].

Current antifungal therapies targeting a broad range of fungal infections, rely primarily on three classes of agents: azoles, polyenes and echinocandins. Azoles, which inhibit ergosterol biosynthesis by targeting the lanosterol 14α-demethylase [8], represent the first line of treatment for aspergillosis, while polyenes such as Amphotericin B (AmB) exert fungicidal activity by directly binding and sequestering ergosterol at the plasma membrane (PM), leading to channel-mediated membrane permeabilization [9]. Despite their clinical utility, these agents are limited by toxicity, drug–drug interactions and increasing azole resistance worldwide [10,11,12,13,14]. Consequently, in 2022, the World Health Organization classified *A. fumigatus* as a critical-priority fungal pathogen [15], emphasizing the urgent need for antifungal agents with novel mechanisms of action that can overcome both antifungal resistance and persistence.

Antimicrobial polymers have emerged as a promising class of next-generation antifungal candidates, offering broad-spectrum activity, and the potential to target microbial membranes through mechanisms distinct from those used by existing drugs, thereby potentially overcoming established resistance mechanisms [16,17,18]. Among these, Polyethylenimine (PEI, Figure S1), a cationic polymer containing repeating ethylenimine units with a high density of primary, secondary and tertiary amine groups [19], has attracted considerable interest as an antibacterial and antifungal agent. The antimicrobial activity of PEI (against *Escherichia coli* and *Staphylococcus aureus* derives from its polycationic character at physiological pH, which enables electrostatic interactions with the negatively charged surfaces of microbial membranes, leading to membrane disruption, permeabilization and, ultimately, cell death [19,20]. In our previous studies, we have demonstrated that hyperbranched polyethyleneimine (PEI) together with its guanidinylated (GPEI) and quaternized (QPEI) derivatives exhibit potent antifungal activity against *A. nidulans*, a well-established model filamentous fungus with broad relevance to the biology and pathogenesis of *Aspergillus* species. Although all three polymers displayed potent antifungal activity with low mammalian cytotoxicity, PEI showed the greatest potency and was therefore selected for mechanistic investigation. Notably, unlike most current antifungals, PEI was active against both quiescent conidia and actively growing germlings [21,22]. Previous mechanistic studies showed that PEI initially accumulates at the fungal plasma membrane before entering the cytoplasm and rapidly induces membrane permeabilization, Reactive Oxygen Species (ROS) production and mitochondrial fragmentation. Given that ergosterol is the principal sterol of the fungal plasma membrane and is essential for membrane fluidity, polarized growth, and membrane trafficking in filamentous fungi [23], while at the same time it is the primary target of polyene antifungals such as AmB [9], alterations in ergosterol organization are considered a potential part of the mechanism of toxicity of PEI.

In addition, we have investigated whether the capacity of hyperbranched PEI to chelate metals could be exploited to synthesize antifungal polymers through coordination with Cu^2+^, a transition metal with well-established antimicrobial properties [24]. Stable PEI-Cu complexes with defined Cu:N molar ratios were synthesized and characterized [22]. PEI-Cu complexes have been shown to retain the antifungal activity of the parent polymer against both quiescent conidia and actively growing germlings of *A. nidulans*, while exhibiting a delayed onset of antifungal activity relative to PEI particularly delaying PEI internalization [22]. The magnitude of this delay increased with copper loading, with the highest copper analogy (PEI-Cu¼) exhibiting the most noticeable delay in antifungal activity and PEI internalization. However, the mechanistic basis of this effect remained unresolved.

Copper homeostasis is tightly regulated in *Aspergillus* species through coordinated uptake, intracellular trafficking and detoxification pathways [25]. While Cu^2+^ is essential for fungal physiology, excess copper promotes oxidative stress and cellular damage [26]. Consequently, fungal copper homeostasis has attracted increasing interest as a potential therapeutic target, including strategies based on copper ionophores or inhibition of copper detoxification pathways [27]. A summary of the current state of knowledge regarding PEI antimicrobial activity and Cu^2+^-mediated modulation of antifungal responses is provided in Table S1.

Although the antifungal properties of PEI-Cu complexes have been characterized to an extent, the mechanism by which free exogenous Cu^2+^ modulates the antifungal activity of PEI has not been established. Furthermore, whether PEI-induced membrane damage is associated with changes in ergosterol lipid organization, similarly to polyene mode of action, has not been investigated. In the present study, we sought to define the mechanism by which exogenous Cu^2+^ modulates PEI antifungal activity in *A. nidulans*, with particular emphasis on the membrane-associated events underlying this response. To achieve this, we examined the concentration and timing of Cu^2+^-mediated protection, assessed whether this protection varies across developmental stages, analyzed the effects of PEI on plasma membrane ergosterol organization, and explored the potential involvement of eisosomes in Cu^2+^-mediated protection against PEI.

## 2. Materials and Methods

### 2.1. Chemicals and Reagents

Hyperbranched polyethylenimine (PEI; average molecular weight 25 kDa, Lupasol^®^ WF, 99%, CAS 9002-98-6), fluorescein isothiocyanate-labelled PEI (PEI-FITC), and the PEI-Cu chelate complex (PEI-Cu¼) were prepared and characterized as previously described [21,22]. Working concentrations were prepared by dilution in standard minimal medium (MM) immediately before use. Amphotericin B (AmB; 500 mg, Cayman Chemical (Ann Arbor, MI, USA), CAS 1397-89-3) was dissolved in DMSO to prepare a concentrated stock solution and diluted in MM to a working concentration of 32 µg mL^−1^ immediately before use. Filipin III (500 µg; Cayman Chemical, CAS 480-49-9) was dissolved in DMSO to prepare a 1 mg mL^−1^ stock solution; stock aliquots were stored at −20 °C protected from light and used within 24 h of preparation. A working solution of 10 µg mL^−1^ filipin III was prepared fresh before each experiment by diluting the stock 1:100 in MM. MitoView™ 650 was purchased from Biotium (Fremont, CA, USA) and used at a final concentration of 0.5 µM as previously described [22]. FM4-64 (100 μg, Invitrogen™, Thermo Fisher Scientific (Waltham, MA, USA), CAS 162112-35-8) was dissolved in DMSO to prepare a 5 mM stock solution. Working solutions (5 µM) were prepared fresh in MM immediately before use. All other chemicals were of analytical grade and obtained from standard commercial suppliers.

### 2.2. A. nidulans Strains, Media and Growth Conditions

Standard minimal medium (MM), complete medium (CM) and *A. nidulans* growth conditions were as described by Cove DJ, 1966 [28]. Unless otherwise stated, growth experiments were performed in MM (pH 6.8) supplemented with 5 mM urea (CAS 57-13-6) as the sole nitrogen and 1% (*w*/*v*) glucose (CAS 50-99-7) as the sole carbon sources. When necessary, supplements were added at adequate concentrations (https://www.fgsc.net/, accessed on 1 August 2026). The auxotrophic strain of *A. nidulans*, *pabaA1* (p-aminobenzoic acid requiring), originating from the wild-type Glasgow FGSC4 strain (https://fungidb.org/fungidb/app/record/dataset/NCBITAXON_227321, accessed on 1 August 2026), was used as a wild-type strain in this study. In brief, the *pabaA1* strain was grown in MM or CM (pH 6.8) supplemented with 5 mM urea as sole nitrogen source, 1% (*w*/*v*) glucose as sole carbon source and 5 mM p-aminobenzoic acid (PABA, CAS Number: 150-13-0). In experiments examining the involvement of eisosomes in the observed phenotypes, a *pilA-gfp* (*pyrG89 pabaA1*) and the *pilAΔ* (*ribo^+^ nkuAΔ* (*argB^+^*) *pyrG89 argB2 pabaB22 riboB2*) strains were used. The latter was supplemented with Uracil (CAS Number: 66-22-8), Uridine (CAS Number: 58-96-8) and PABA as required (https://www.fgsc.net/). Conidial suspensions were prepared from colonies grown on CM plates for 3–4 days at 37 °C; conidia were harvested in 0.05% (*v*/*v*) Tween-20 (CAS Number: 9005-64-5) in sterile water, filtered through Miracloth (EMD Millipore (Burlington, MA, USA)), and counted using a Neubauer hemocytometer.

For microscopy experiments involving germlings, approximately 10^6^ *pabaA1* conidiospores mL^−1^ were inoculated in 2 mL of MM (supplemented with 5 mM urea, 1% (*w*/*v*) glucose and 5 mM PABA) in 35 mm Petri dishes containing a glass coverslip, and incubated at 25 °C for 12–14 h to obtain growing germlings. Short exposure times with PEI (10–60 min) were selected to capture the immediate, early-stage chronological events of PEI-induced damage. Copper-depleted MM (MM − Cu) was prepared by simply omitting Cu from the MM recipe.

### 2.3. Microscopy

#### 2.3.1. Epifluorescence and Phase Contrast Microscopy

Coverslips containing the attached hyphal germlings of the *pabaA1* strain were observed under an Axioplan 2 Observer Z1 fluorescence microscope (Carl Zeiss, Jena, Germany) equipped with a Plan-Apochromat 40× or 100×/1.40 NA oil immersion objective lens and appropriate fluorescent light filter sets. Images were captured with a digital camera (Retiga 2000R, QImaging, Surrey, BC, Canada) and Occular version 2.01 acquisition software (Teledyne Photometrics, Inc., Tucson, AZ, USA) and were processed with Fiji ImageJ software (version 1.54f; National Institutes of Health, Bethesda, MD, USA) [29]. For each experiment, all images were acquired using identical microscope settings (objective, exposure time, gain and illumination intensity) for all conditions within the same experiment. Images were processed identically for display purposes.

#### 2.3.2. Confocal Laser Microscopy (CLM)

10^6^ *pabaA1* conidia mL^−1^ in 0.05% (*v*/*v*) Tween-20 were cultured in MM supplemented with 5 mM urea and 1% (*w*/*v*) glucose as the sole nitrogen and carbon sources, respectively, in 8-well µ-Slides (Ibidi GmbH, Gräfelfing, Germany). Cells were observed using a Leica TCS SP5 (Leica Microsystems Ltd., Milton Keynes, UK) confocal microscope with a Leica HCX PL APO CS 63×/1.4 NA oil immersion lens. FITC was excited with a 496 nm laser. Images were acquired and processed using Fiji ImageJ software (version 1.54f), as described in Athanasopoulos et al., 2015 [30].

### 2.4. Copper Treatment

#### 2.4.1. Cu^2+^ Co-Treatment

For co-treatment experiments, Cu^2+^ (as CuSO_4_), at concentrations specified in each experiment was added simultaneously with PEI (5 µg mL^−1^) to the growth medium. The PEI-Cu¼ was used as a PEI-Cu chelate control, applied at a final concentration of 5 µg mL^−1^ of the preformed PEI-Cu complex, whenever necessary as previously described [22].

#### 2.4.2. Cu^2+^ Pre-Treatment

For conidial pre-treatment experiments, approximately 10^5^ *pabaA1* conidia mL^−1^ were resuspended in MM supplemented with 160 µM CuSO_4_ (MM + Cu) or in MM − Cu and incubated at 37 °C for 3 h. Residual extracellular Cu^2+^ was then removed by successive centrifugation at 13,000 rpm (~22.500× *g*), using an RA-159G rotor (Kubota 7780 centrifuge, KUBOTA Corporation, Tokyo, Japan), for 20 min and washes with 2 mL of ddH_2_O for a total of two cycles. The washed conidial pellet was then resuspended in MM with PEI (5 µg mL^−1^) and the appropriate auxotrophies and incubated at 37 °C for a further 4 h period before a colony forming unit (CFU) assay (see Section 2.5).

#### 2.4.3. Cu^2+^ Pre-Treatment of Germlings

For germling pre-treatment experiments, early *pabaA1* germlings ~11 h grown on coverslips were incubated with Cu^2+^ (at concentrations ranging from 1.6 to 480 µM as specified) in MM for 3 h at 25 °C. This concentration range was selected to span from physiological (1.6 μM) to the maximum non-cytotoxic Cu^2+^ concentration (480 µM); concentrations above 480 µM were not used due to endogenous Cu^2+^ cytotoxicity. Following pre-treatment, the Cu^2+^-containing medium was removed and the coverslips were washed twice with 2 mL MM to remove residual extracellular Cu^2+^. MM containing PEI or PEI-FITC (5 µg mL^−1^) was then added and cells were incubated for a further 1–2 h period at 25 °C before staining and microscopic observation. Untreated control germlings not receiving PEI treatment were kept at 4 °C for the equivalent 1–2 h period to minimize additional growth while controlling for incubation duration.

#### 2.4.4. Post-Treatment with Cu^2+^

For post-treatment with Cu^2+^ experiments, germlings were first exposed to PEI or PEI-FITC (5 µg mL^−1^), followed by Cu^2+^ (160 µM) addition at selected time points. In the experiment about the Cu^2+^ effect on PEI-induced mitochondrial fragmentation, Cu^2+^ was added at 5, 10 or 15 min after PEI addition and incubated for a further 15 min before MitoView staining and imaging. In the time-course confocal experiment, Cu^2+^ was added at the 60 min time point, after mitochondrial network fragmentation had already been established, and cells were observed for a further 30 min. In FM4-64 experiments, Cu^2+^ was added for 5 min after the 10 min PEI treatment before the FM4-64 dye loading.

### 2.5. Colony-Forming Unit (CFU) Survival Assay

Conidial survival was assessed by CFU assay as previously described [21,22] with slight modifications to incorporate Cu^2+^ treatment conditions. Briefly, approximately 10^5^ *pabaA1* conidia mL^−1^ (or the indicated strains for eisosome experiments) were resuspended in 0.05% (*v*/*v*) Tween-20 and incubated with PEI (5 µg mL^−1^) alone or together with Cu^2+^ at the indicated concentrations (Section 2.3), in standard MM or MM − Cu, for 4 h at 37 °C. Following incubation, each sample was diluted 1:10 in 0.01% (*v*/*v*) Tween-20, and 100 µL was plated onto CM plates to yield approximately 300 colonies per plate. This dilution, together with dispersion on CM agar following plating, reduced the effective PEI concentration to well below its minimal inhibitory concentration (5 µg mL^−1^) during colony establishment. Plates were incubated for 1–2 days at 37 °C and colonies were counted. Relative conidial survival was expressed as the percentage of colonies relative to the corresponding untreated control (set to 100%). Three technical replicates were performed per condition per experiment, and experiments were independently repeated at least three times (*n* ≥ 3).

### 2.6. Mitochondrial Network Morphology Assay

Mitochondrial network morphology was assessed by MitoView™ 650 staining as previously described [22], with slight modifications to incorporate Cu^2+^ co-treatment conditions. Briefly, *pabaA1* (or *pilAΔ*/*pilA-gfp* strains as specified) conidiospores were grown on coverslips in 35 mm Petri dishes in MM for 14 h at 25 °C. PEI (5 µg mL^−1^) was then added alone or simultaneously with Cu^2+^ at the indicated concentrations and cells were incubated for 15 min at RT (Room Temperature; approximately 23–25 °C). MitoView™ 650 (0.5 µM) was added to the medium at least 15 min prior to imaging and cells were maintained in the dark. For post-treatment with Cu^2+^ experiments, MitoView™ 650 staining was performed for 15 min at RT, in the dark, followed by PEI treatment and finally Cu^2+^ (160 µM) was added at 5, 10 or 15 min after PEI and incubated for 15 min before observation. Coverslips were mounted on microscope slides and immediately observed using epifluorescence microscopy (Axioplan 2 Observer Z1 fluorescence microscope, Carl Zeiss, Jena, Germany). The mitochondrial network of each cell was classified as tubular/filamentous (interconnected network) or fragmented (majority of mitochondrial network appeared as short punctate or discontinuous structures [22]), and the percentage of cells displaying a tubular mitochondrial network was calculated. Approximately 200 cells were scored per sample, and each experiment was independently repeated at least three times (*n* ≥ 3; total ≥ 600 cells scored per condition).

### 2.7. Time Course CLM

For the time-course CLM chase experiment, *pabaA1* conidiospores were grown as described in Section 2.3.2 for 14 h at 25 °C. Cells were stained with MitoView™ 650 (0.5 µM, added at least 15 min prior to imaging) and PEI-FITC (5 µg mL^−1^) was added to the medium. Time-course images were captured every 10 min over a 90 min observation period. PEI-FITC was excited using a 496 nm laser. Differential Interference Contrast (DIC), MitoView™ 650 (red) and PEI-FITC (green) channels were recorded simultaneously. Cu^2+^ (160 µM) was added to the imaging chamber at the 50 min time point, after mitochondrial network fragmentation and vacuolization had been observed to occur (typically at 40–50 min). Images were acquired and processed using Fiji ImageJ software (version 1.54f).

### 2.8. FM4-64 Endocytic Trafficking Assay

To assess PEI-induced changes in plasma membrane permeability and endocytic trafficking kinetics, the lipophilic styryl dye FM4-64 was used as an endocytic tracer. *pabaA1* conidiospores were grown on glass coverslips in 35 mm Petri dishes in MM (supplemented with 5 mM urea, 1% (*w*/*v*) glucose and 5 mM PABA) for 14 h at 25 °C to obtain actively growing germlings. Where indicated, Cu^2+^ pre-treatment or delayed Cu^2+^ addition was applied as described in Section 2.4.3 and Section 2.4.4. For co-treatment, Cu^2+^ (160 µM) was added simultaneously with PEI. Following the specified treatment period (10 min at RT), the growth medium was removed and 200 µL of ice-cold FM4-64 working solution (5 μM) was applied directly to the coverslip. Cells were incubated on ice for 15 min in the dark to allow surface labelling while minimizing dye internalization. The coverslip was then washed twice with ice-cold MM to remove unbound dye, and room-temperature MM was immediately added. Cells were observed using epifluorescence microscopy at 2.5 min after the addition of the room-temperature MM to capture early FM4-64 localization (plasma membrane located/early endosomal). FM4-64 distribution was classified as on the plasma membrane or internalized.

### 2.9. Filipin III Ergosterol Staining

To assess the subcellular distribution of ergosterol in response to PEI treatment, filipin III staining was performed as follows. *pabaA1* conidiospores were grown on glass coverslips in 35 mm Petri dishes in MM (supplemented with 5 mM urea, 1% (*w*/*v*) glucose and 5 mM PABA) for 12–14 h at 25 °C. AmB (32 µg mL^−1^), PEI (5 µg mL^−1^) alone, or PEI (5 µg mL^−1^) in combination with Cu^2+^ (160 µM; co-treatment) were added to the medium and cells were incubated for 30 min at room temperature. Following treatment, the medium was carefully removed and 200 µL of freshly prepared filipin III working solution (10 µg mL^−1^ in MM, without additional Cu^2+^) was applied directly to the coverslip. Cells were stained for 5 min at room temperature in the dark. The filipin III solution was then removed and the coverslip was washed twice with 2 mL MM; residual MM was left on the coverslip to prevent dryness. Cells were immediately observed under a UV/blue excitation filter (DAPI channel) by epifluorescence microscopy. Filipin III fluorescence distribution was assessed qualitatively in terms of subcellular localization pattern (plasma membrane-restricted, intracellular vesicular/punctate, or polarized) in at least 100 cells per condition across three independent experiments.

### 2.10. Statistical Analysis

All experiments were independently repeated at least three times unless otherwise stated. Data are presented as mean ± standard deviation (SD). Statistical significance was assessed using an unpaired two-tailed Student’s *t*-test for pairwise comparisons. Significance thresholds: *, *p* < 0.05; **, *p* < 0.01; ***, *p* < 0.001; ****, *p* < 0.0001; ns, not significant. Statistical analyses were performed using GraphPad Prism (GraphPad Prism version 8.0.0; GraphPad Software, San Diego, CA, USA).

A biological replicate was defined as an independently performed experiment initiated from a separate conidial culture on a different day. A technical replicate was defined as repeated measurements or platings performed from the same biological sample. Statistical analyses were performed using the biological replicates, while technical replicates were averaged to generate a single value for each biological replicate. For microscopy-based phenotypic analyses, at least 100 cells from randomly selected fields were analyzed per condition in three independent biological experiments, with phenotype percentages calculated separately for each biological replicate.

### 2.11. Control Treatments

For all experiments, parallel CuSO_4_-only controls (160 μM of Cu^+2^) were included under identical incubation conditions. Unless otherwise stated, Cu^2+^ treatment alone did not produce detectable changes compared with untreated controls.

## 3. Results

### 3.1. Intracellular Cu^2+^ Rescues Conidia from PEI-Induced Loss of Viability in a Concentration-Dependent Manner

**Figure 1:**
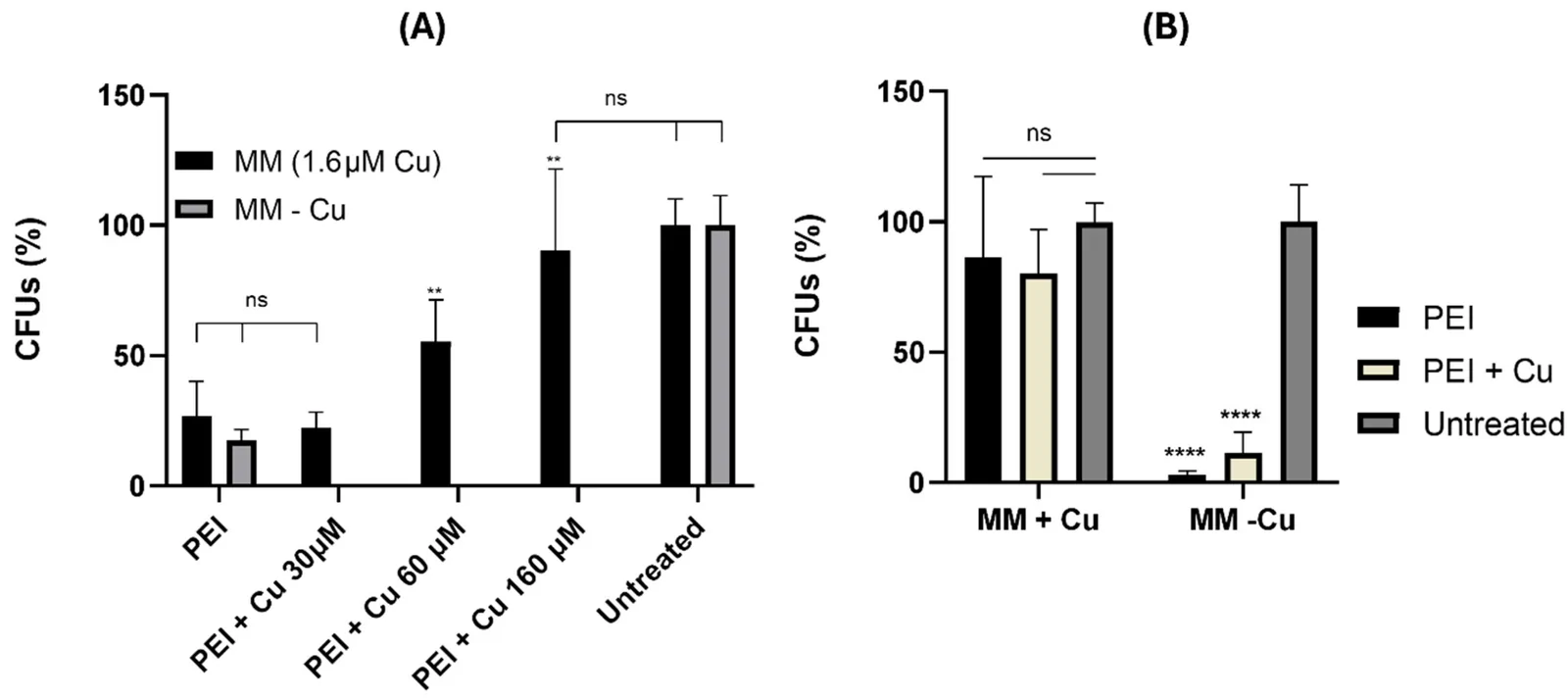
Exogenous and intracellular Cu^2+^ protect *A. nidulans* conidia from PEI-induced death. (**A**) Conidial survival (CFU, % of untreated control) following 3 h co-treatment with PEI (5 µg mL^−1^) and Cu^2+^ (30, 60 or 160 µM) in standard minimal medium MM (1.6 μM of Cu) or Cu^2+^-depleted minimal medium (MM − Cu) at 37 °C. Co-treatment with Cu^2+^ increased conidial survival in a concentration-dependent manner, whereas conidia treated with PEI in MM − Cu exhibited similar survival with those in standard MM. Untreated controls in MM and MM − Cu are shown. (**B**) Conidial survival (CFU, % of untreated control) following 4 h pre-incubation in standard MM supplemented with 160 µM Cu^2+^ (MM + Cu) or MM − Cu at 37 °C, followed by washout of extracellular Cu^2+^ and subsequent 4 h treatment with PEI (5 µg mL^−1^). Pre-incubation in MM + Cu was associated with substantially greater conidial survival following PEI treatment than pre-incubation in MM − Cu. Data are expressed as a percentage of the corresponding untreated control. Parallel CuSO_4_-only controls (160 μM) were included for all experiments shown. Under the conditions examined, CuSO_4_ treatment alone did not produce detectable differences from untreated controls. Bars represent mean ± SD of 6 independent biological replicates (*n* = 6), 3 technical replicates each and stars represent statistical significance in comparison to the respective controls. Statistical significance was determined using an unpaired two-tailed Student’s *t*-test. Unless otherwise indicated by connecting lines, all statistical comparisons were made relative to PEI (**A**) or the corresponding untreated control (**B**). *p* < 0.01 (**), *p* < 0.0001 (****) and ns; not significant.

To determine whether non-chelated, exogenous Cu^2+^ modulates the antifungal activity of Polyethylenimine (PEI) against *A. nidulans*, we performed colony-forming unit (CFU) survival assays on quiescent conidiospores, in the presence of PEI (5 µg mL^−1^ which corresponds to MIC of PEI [22] as determined using a slightly modified EUCAST DEF 9.3.1 method [31]) and increasing concentrations of Cu^2+^ in standard minimal medium (MM) or copper-depleted MM (MM − Cu). We examined Cu^2+^ concentrations as multiples of ten (16, 160 μM) of the physiological level in MM (1.6 µM), including intermediate concentrations (4.8, 30 and 60 µM), to better define the threshold for protective effects. In standard MM, co-treatment with Cu^2+^ resulted in a concentration-dependent increase in conidial survival with substantial rescue at 60 µM, and near-complete protection at 160 µM compared to the untreated control (Figure 1A). No significant rescue of PEI-induced loss of viability was observed at physiological or modestly elevated Cu^2+^ concentrations (1.6, 4.8, 16, or 30 µM) and for clarity, data for 4.8 and 16 µM Cu^2+^ are omitted from the graph because they showed no significant difference from the 1.6 or 30 μM concentrations. Notably, PEI-treated conidia in MM without copper exhibited similar survival to those in standard MM, indicating that only Cu^2+^ concentrations above physiological levels provide effective protection against PEI-induced loss of viability.

To determine whether Cu^2+^-mediated protection requires the continuous extracellular presence of the ion or whether pre-acquired intracellular Cu^2+^ is sufficient, we pre-incubated conidia in standard MM or MM without the standard 1.6 µM Cu^2+^ for 4 h prior to PEI exposure, followed by bulk removal of extracellular Cu^2+^ before PEI supplementation. Pre-incubation with Cu at 160 μM fully protected conidia from PEI-induced death, whereas pre-incubation in the absence of Cu resulted in near-complete loss of viability upon PEI treatment (Figure 1B). These results demonstrate that an intracellular pool of Cu^2+^ accumulated prior to PEI treatment is sufficient to reduce PEI-induced loss of viability, and that the Cu^2+^ content of MM is a critical determinant of their susceptibility to PEI.

### 3.2. Cu^+2^-Mediated Protection from PEI-Induced Loss of Viability Is Independent of Eisosome Assembly

**Figure 2:**
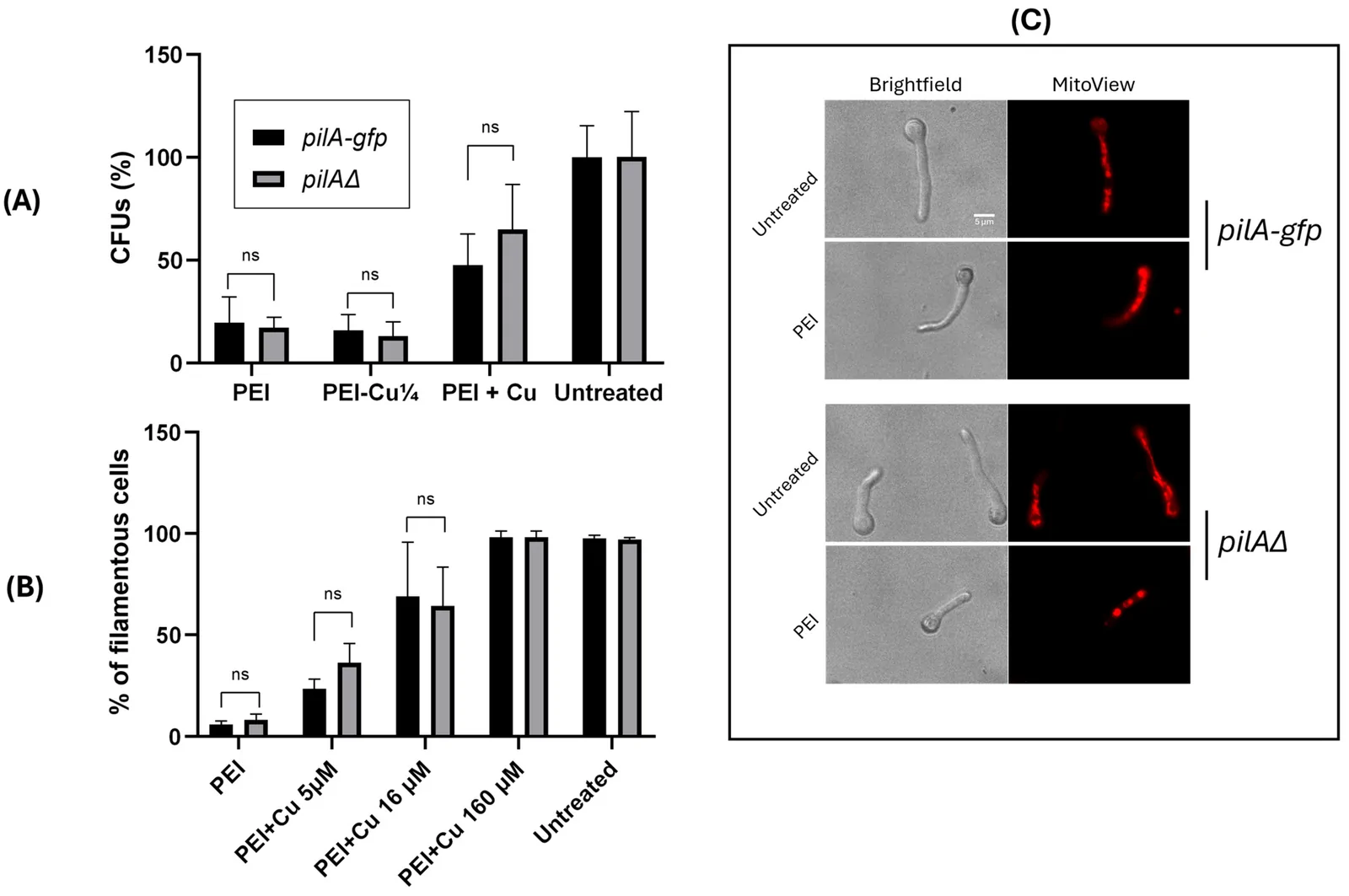
(**A**) Cu^2+^-mediated protection against PEI-induced loss of viability is independent of eisosome assembly. Conidia survival (CFU, % of untreated cells) of *A. nidulans pilA-gfp*, (grey) and the eisosome-lacking *pilAΔ* (black) strains, following 4 h treatment with PEI (5 µg mL^−1^) at 37 °C, PEI-Cu chelate complex (PEI-Cu¼), PEI + Cu^2+^ (160 µM, co-treatment), or untreated cells. Treatment with Cu^2+^ increased conidial survival in both strains. (**B**) Effect of Cu^2+^ on PEI-induced mitochondrial changes. Percentage of cells retaining a filamentous (tubular) mitochondrial network following 15 min co-treatment of 14 h germlings with PEI alone (5 µg mL^−1^) at RT or with the addition of Cu^2+^ (4.8, 16 or 160 µM), or untreated cells. *pilA-gfp* and *pilAΔ* cells stained with MitoView 650 are compared. Cu^2+^ restored mitochondrial network integrity in a concentration-dependent manner in both strains. Bars represent mean ± SD of ≥3 biological replicates. (**C**) Representative fluorescence microscopy images of mitochondrial network morphology in untreated and PEI-treated *A. nidulans* germlings of the *pilA-gfp* and *pilAΔ* strains following staining with MitoView 650. Untreated germlings exhibit the characteristic filamentous mitochondrial network, whereas PEI treatment induces mitochondrial fragmentation in both strains, indicating that loss of PilA does not alter the mitochondrial phenotype caused by PEI. Parallel CuSO_4_-only controls (160 μM) were included for all experiments shown. Under the conditions examined, CuSO_4_ treatment alone did not produce detectable differences from untreated controls. Scale bar 5 μm, ns; not significant.

Having established that exogenous Cu^2+^ markedly rescues *A. nidulans* conidia from PEI-induced loss of viability, we investigated the cellular mechanism underlying this protective effect. Given that eisosomes are conserved plasma membrane-associated compartments implicated in plasma membrane organization and fungal stress adaptation [30,32], and elevated Cu^2+^ induces eisosome formation in *A. nidulans* [Sophianopoulou and Athanasopoulos, unpublished observations], we assessed whether intact eisosomes are required for Cu^2+^-mediated protection. To determine that, we compared both conidial survival and mitochondrial network integrity between a *pilA-gfp* strain and an eisosome-deficient *pilAΔ* mutant, (see materials and methods), following co-treatment with PEI (5 µg mL^−1^) and Cu^2+^ (160 µM). CFU assays revealed that the co-treatment produced a comparable and substantial increase in conidial survival in both the *pilA-gfp* and *pilAΔ* strains relative to PEI alone (Figure 2A). To distinguish the effect of free Cu^2+^ from that of Cu^2+^ coordinated to PEI, we also included the previously characterized PEI-Cu chelate (PEI-C¼, [22]). The latter did not markedly increase survival in comparison to PEI alone in either strain under the examined conditions, consistent with our previous findings demonstrating that Cu^2+^ chelation to PEI delays but does not fully abolish its antifungal activity [22].

To extend our analysis to a subcellular level, we assessed mitochondrial network morphology using MitoView staining across a range of Cu^2+^ concentrations (4.8, 16 and 160 µM) co-administered with PEI. In both *pilA-gfp* and *pilAΔ* strains, PEI alone resulted in extensive mitochondrial network fragmentation and Cu^2+^ co-treatment recovered mitochondrial network integrity in a concentration-dependent manner, with full restoration at 160 µM (Figure 2B). The identical dose–response profiles observed for the *pilA-gfp* and *pilAΔ* strains across both readouts indicate that the Cu^2+^-dependent rescue of PEI-induced loss of viability does not require the eisosome-organizing protein PilA, and is therefore unlikely to be mediated through eisosome-associated plasma membrane compartmentalization or similar pathways.

### 3.3. Exogenous Cu^2+^ Attenuates PEI-Induced Alterations in Plasma Membrane Trafficking in Germlings

**Figure 3:**
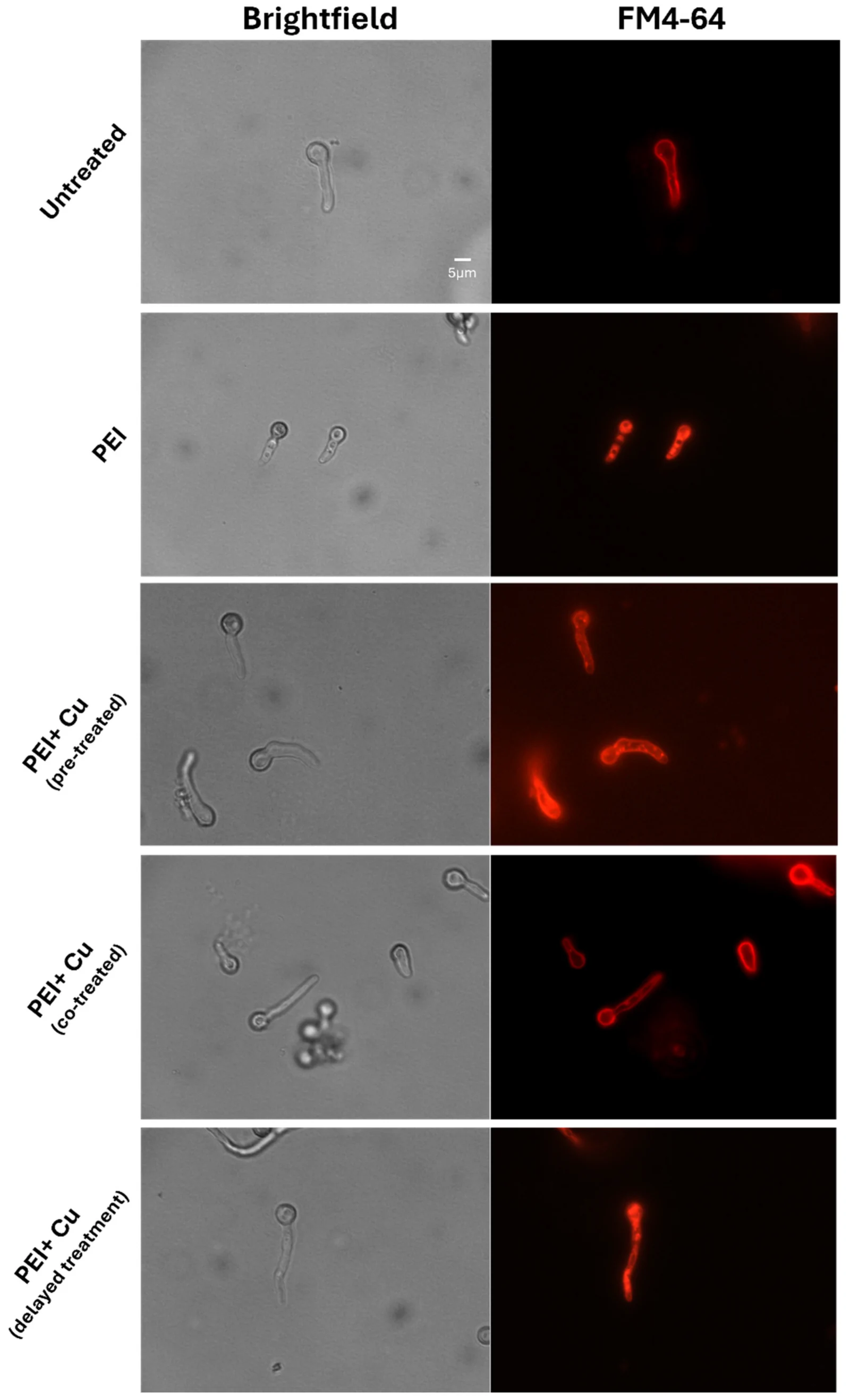
Cu^2+^-dependent attenuation of PEI-induced plasma membrane trafficking alterations. Representative fluorescence microscopy images of untreated, PEI-treated, and PEI + Cu^2+^-treated 14 h germlings stained with FM4-64 and observed after 2.5 min at RT. At this time point, FM4-64 is primarily associated with the plasma membrane, while internalization has just begun. PEI (5 μg/mL) induces acute FM4-64 internalization to its final subcellular distribution, whereas co-treatment with Cu^2+^ (160 μM) prevents this phenotype and FM4-64 localization kinetics resemble those of untreated cells. Cu^2+^ pre-treatment (160 μM, 3 h) followed by its removal resulted in an intermediate phenotype, with FM4-64 localized both at the plasma membrane and in early endosomes. In contrast, Cu^2+^ addition following PEI treatment resembles the PEI-induced FM4-64 internalization phenotype. At least 100 cells were scored per condition across three independent experiments. Parallel CuSO_4_-only controls (160 μM) were included for all experiments shown. Under the conditions examined, CuSO_4_ treatment alone did not produce detectable differences from untreated controls.

To investigate the upstream effect of PEI on plasma membrane trafficking, we monitored the internalization kinetics of FM4-64, a styryl dye that traces the endocytic route from the plasma membrane to vacuolar compartments in filamentous fungi, in 14 h germlings following PEI treatment with or without Cu^2+^. Under control conditions, FM4-64 was loaded for 15 min on ice and allowed to internalize at room temperature; at 2.5 min, fluorescence remained predominantly at the plasma membrane and early endosomal structures, consistent with normal endocytic kinetics [33]. In cells treated with PEI (5 µg mL^−1^, 10 min prior to dye loading), FM4-64 signal was already strongly internalized and associated with vacuolar compartments within 2.5 min, accelerates FM4-64 internalization, consistent with our previous demonstration that PEI rapidly compromises plasma membrane integrity [21,22]. When Cu^2+^ (160 µM) was added simultaneously with PEI, FM4-64 internalization kinetics resembled those of untreated control cells, demonstrating that Cu^2+^ prevents PEI-induced acceleration of endocytic trafficking (Figure 3). Cu^2+^ pre-treatment (160 µM, 3 h, 25 °C), followed by bulk removal of extracellular Cu^2+^ before PEI treatment, also affected FM4-64 internalization, though less effectively than simultaneous co-treatment. This intermediate phenotype suggests that pre-acquired intracellular Cu^2+^ partially protects against or delays the early membrane-associated effects of PEI. In contrast, addition of Cu^2+^ after PEI treatment did not restore normal FM4-64 kinetics, indicating that Cu^2+^ cannot reverse PEI-induced membrane trafficking alterations once it has occurred.

### 3.4. PEI Induces Redistribution of Ergosterol from the Plasma Membrane and Causes Polarized Ergosterol Accumulation at the Hyphal Tip

**Figure 4:**
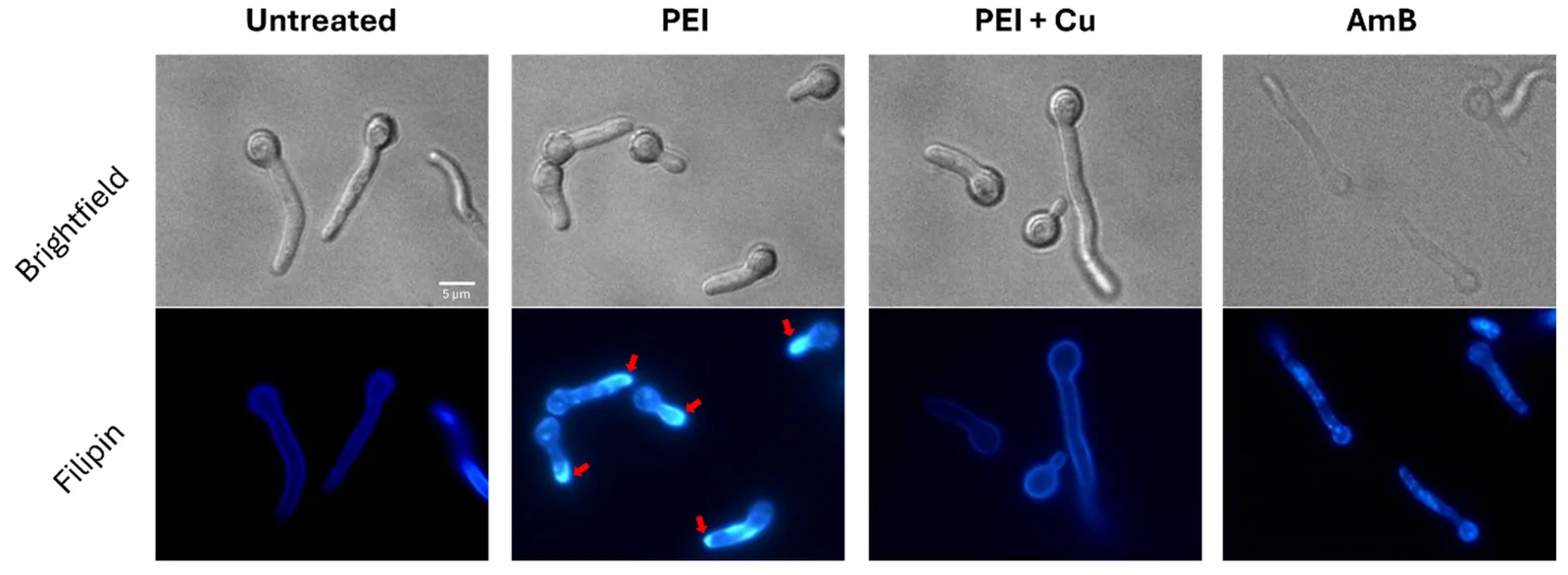
PEI-induced redistribution of ergosterol from the plasma membrane and polarized accumulation at the hyphal tip. Representative epifluorescence images from filipin III staining of ergosterol distribution in *A. nidulans* germlings. Germlings (12–14 h, 25 °C) were either untreated, treated with PEI (5 µg mL^−1^), co-treated with PEI + Cu^2+^ (160 µM) or AmB (32 µg mL^−1^) for 30 min at RT, stained with filipin III (10 µg mL^−1^ in MM, 5 min, RT) and observed under a DAPI/blue filter. In untreated cells, filipin signal localizes to the plasma membrane. Cells were qualitatively sorted in three different categories: (1) plasma membrane localization, (2) intracellular vesicular/punctate staining, (3) intracellular vesicular/punctate structures plus hyphal-tip accumulation. In 91 ± 4% of AmB-treated germlings, filipin is redistributed to intracellular vesicular/punctate structures. In 90 ± 6% of PEI-treated germlings, filipin is similarly detected in the cell interior and additionally accumulates preferentially at the hyphal tip (red arrowheads). Co-treatment with Cu^2+^ prevented PEI-induced redistribution of filipin in 93 ± 6% of germlings maintaining a staining pattern similar to that of untreated controls. The staining patterns shown were consistently observed across randomly selected fields (4–6 cells/field) for each condition tested in three independent biological experiments, with at least 100 cells analyzed per condition. Parallel CuSO_4_-only controls (160 μM) were included for all experiments shown. Under the conditions examined, CuSO_4_ treatment alone did not produce detectable differences from untreated controls.

To investigate whether PEI disrupts the organization of the fungal PM due to its interaction with lipids as do other antifungals, we examined the distribution of ergosterol, the main sterol component of the plasma membrane in *A. nidulans* germlings using Filipin III, a fluorescent polyene that specifically binds to ergosterol. AmB, which exerts its antifungal activity through direct ergosterol sequestration at the PM, was used as a positive control (32 µg mL^−1^, 30 min, RT). In untreated germlings, filipin fluorescence was confined to the plasma membrane, consistent with the established homeostatic localization of ergosterol (Figure 4, untreated cells). AmB treatment caused marked redistribution of filipin signal from the PM to the cell interior, appearing as discrete vesicular puncta and larger intracellular structures, confirming reliably ergosterol displacement from the PM (Figure 4, AmB).

**Figure 5:**
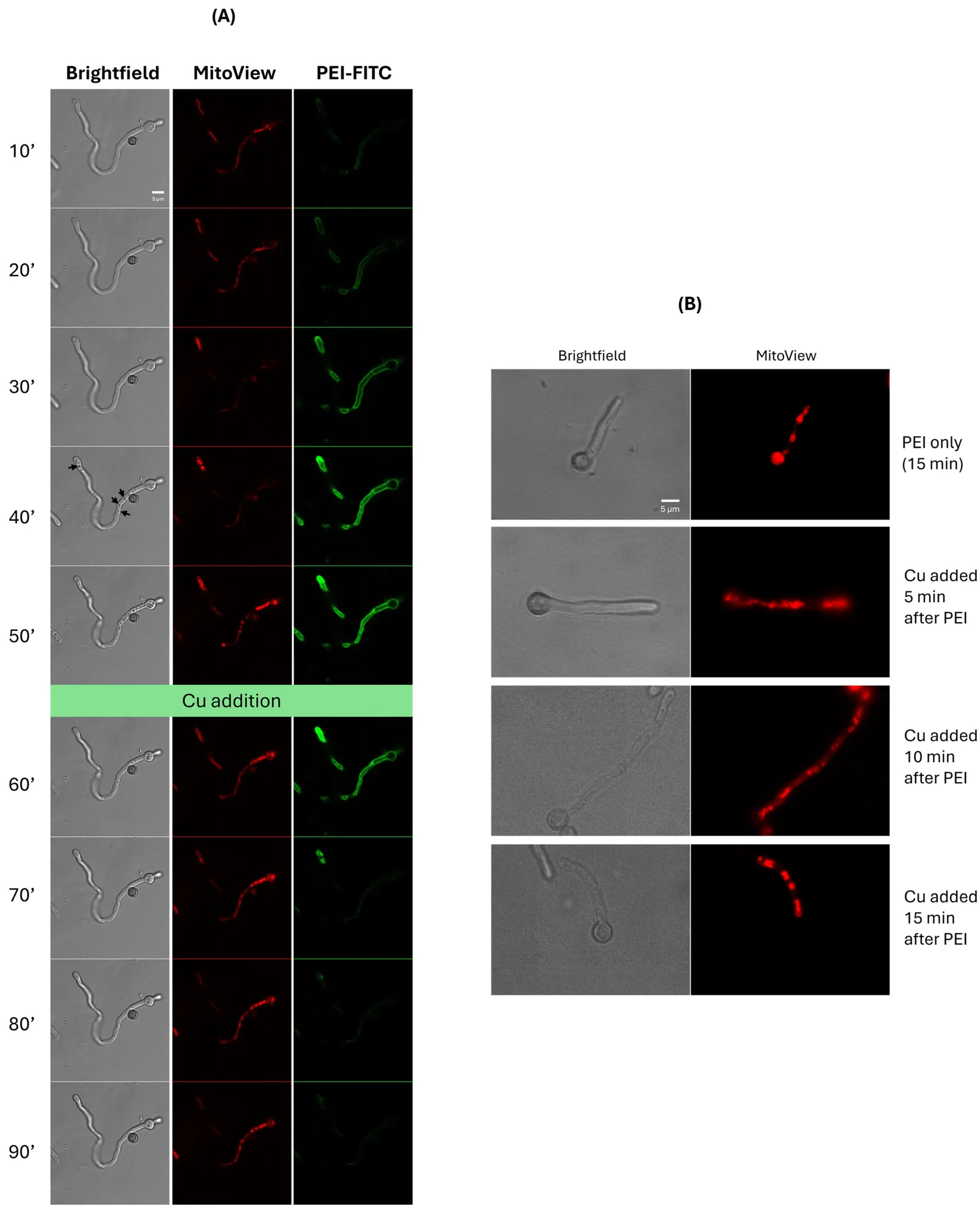
Cu^2+^ effect on PEI-induced mitochondrial fragmentation. (**A**) Confocal fluorescence microscopy time course images of 14 h germlings treated with PEI (5 µg mL^−1^), following staining with MitoView, and observed for 90 min while Cu^2+^ was added at 60 min. Mitochondrial network integrity, PEI fluorescence as well as brightfield morphological changes were observed. PEI acts by causing mitochondrial fragmentation, which is visible at 50 min while intense vacuolization is observed at approximately 40 min. Copper added after fragmentation was observed to examine potential effects on already fragmented mitochondria. Adding Cu^2+^ causes fading of PEI fluorescence, reverses the vacuolization observed on brightfield (fully restored at 70 min) but does not affect the mitochondrial fragmentation. (**B**) Representative epifluorescence images of germlings following treatment with PEI (5 µg mL^−1^). Cu^2+^ (160 µM) was added 5, 10 or 15 min after PEI addition and incubation continued for a further 15 min before microscopic observation. Germlings treated with PEI alone were observed after 15 min. Early Cu addition (5 min after PEI treatment) largely preserves mitochondrial network integrity and prevents mitochondrial fragmentation. In contrast, delayed Cu^2+^ addition results in progressively reduced protection, with the mild mitochondrial fragmentation observed when Cu^2+^ was added after 10 min of PEI exposure and the extensive fragmentation observed after 15 min. Brightfield and MitoView 650 (red) channels are shown. Black arrows indicate points of intense vacuolization observed at the brightfield in the presence of PEI.

In germlings treated with PEI (5 µg mL^−1^, 30 min, RT), filipin fluorescence was likewise detected in the cell interior as vesicular/punctate intracellular structures qualitatively similar to those observed in AmB-treated cells, indicating that PEI also causes redistribution of ergosterol away from the PM (Figure 4). However, PEI-treated germlings additionally displayed a distinct spatial pattern not observed in untreated or AmB-treated cells: filipin fluorescence was markedly enriched at the hyphal tip (Figure 4, arrowheads). This polarized ergosterol distribution closely resembled the subcellular localization pattern of PEI-FITC in germlings, in which polymer fluorescence similarly accumulated preferentially at the hyphal tip (see Figure 5A 40 and 50 min), suggesting that ergosterol redistribution occurs preferentially at sites of active PEI accumulation. In contrast, co-treatment with Cu^2+^ (160 µM) prevented the PEI-induced redistribution of filipin fluorescence. Filipin fluorescent signal remained mainly on the plasma membrane, resembling the staining pattern observed in untreated germlings, while the characteristic intracellular puncta and increased fluorescence on the hyphal tip induced by PEI were no longer evident (Figure 4, PEI + Cu^2+^).

Population-level quantification revealed that upon PEI exposure, 90 ± 6% (mean ± SD) of the examined germlings exhibited filipin internalization (vesicular/punctate intracellular structures) plus accumulation at their tips. A similarly high proportion of AmB-treated germlings (91 ± 4%) exhibited redistribution of filipin staining from the plasma membrane to intracellular vesicular/punctate structures, although without the characteristic hyphal-tip accumulation observed following PEI treatment. In contrast, when Cu^2+^ (160 µM) was supplemented simultaneously with PEI, approximately 93 ± 6% of germlings retained the normal membrane-localized phenotype similar to that observed in untreated controls.

### 3.5. Time-Dependent Rescue of PEI-Induced Mitochondrial Fragmentation by Cu^2+^

To determine whether Cu^2+^ can affect established PEI-induced mitochondrial damage or whether its protective action is restricted to an early window preceding fragmentation, we performed time-course confocal microscopy of 14 h germlings treated with PEI-FITC and stained with the mitochondrial marker MitoView 650. In the absence of Cu^2+^, PEI-FITC progressively accumulated in the cell interior over 10–50 min, accompanied by the development of extensive mitochondrial network fragmentation and vacuolization, both of which were apparent by 40–50 min of exposure (Figure 5A). When Cu^2+^ (160 µM) was added at the 60 min time point, selected because previous time-course experiments demonstrated that PEI-induced mitochondrial fragmentation was already established before this stage, the fragmented mitochondrial phenotype remained over the subsequent 30 min of observation (Figure 5A, post-Cu addition panels at 60–90 min). However, Cu^2+^ addition produced two notable effects: a time-dependent reduction in detectable PEI-FITC fluorescence, and a progressive time-limited attenuation of the initial cellular perturbations due to PEI-induced vacuolization, which was visible by brightfield microscopy of PEI-induced vacuolization visible by brightfield microscopy. Specifically, vacuolization appeared at 40 min and was fully restored at 70 min. These observations indicate that Cu^2+^ fails to restore mitochondrial network integrity once fragmentation has occurred, despite being associated with reduced PEI-FITC fluorescence and a gradual attenuation of PEI-induced vacuolization.

To precisely define the temporal window within which Cu^2+^ protection remains effective, we performed a complementary experiment in which Cu^2+^ (160 µM, 15 min) was introduced at 5, 10 or 15 min post-PEI administration, and mitochondrial network morphology was assessed thereafter. Cu^2+^ addition at 5 min after PEI treatment largely preserved the filamentous mitochondrial network; addition at 10 min resulted in intermediate protection; and addition at 15 min was insufficient to prevent extensive mitochondrial fragmentation (Figure 5B). Together, these results suggest that Cu^2+^ acts upstream of, or during the early stages of the cascade of PEI-induced cellular damage leading to mitochondrial fragmentation, with protection observed only when Cu^2+^ was added during approximately the first 5–10 min following PEI exposure.

Since a consistent reduction in PEI-FITC fluorescence was observed following Cu^2+^ addition, we investigated whether this effect was specific to growing germlings or represented a general response to Cu^2+^ exposure. As Cu^2+^ addition consistently reduced the detectable fluorescence of PEI-FITC, this observation cannot be used to distinguish between changes in PEI accumulation or retention and Cu^2+^-dependent fluorescence quenching. Although the mechanistic basis of this observation remains unclear, it supports the hypothesis that Cu^2+^ interferes with the accumulation or retention of PEI at the plasma membrane or within the fungal cell (discussed in Section 4).

### 3.6. Cu^2+^ Pre-Treatment Protection Against PEI-Induced Loss of Viability Depends on the Developmental Stage of the Cells

**Figure 6:**
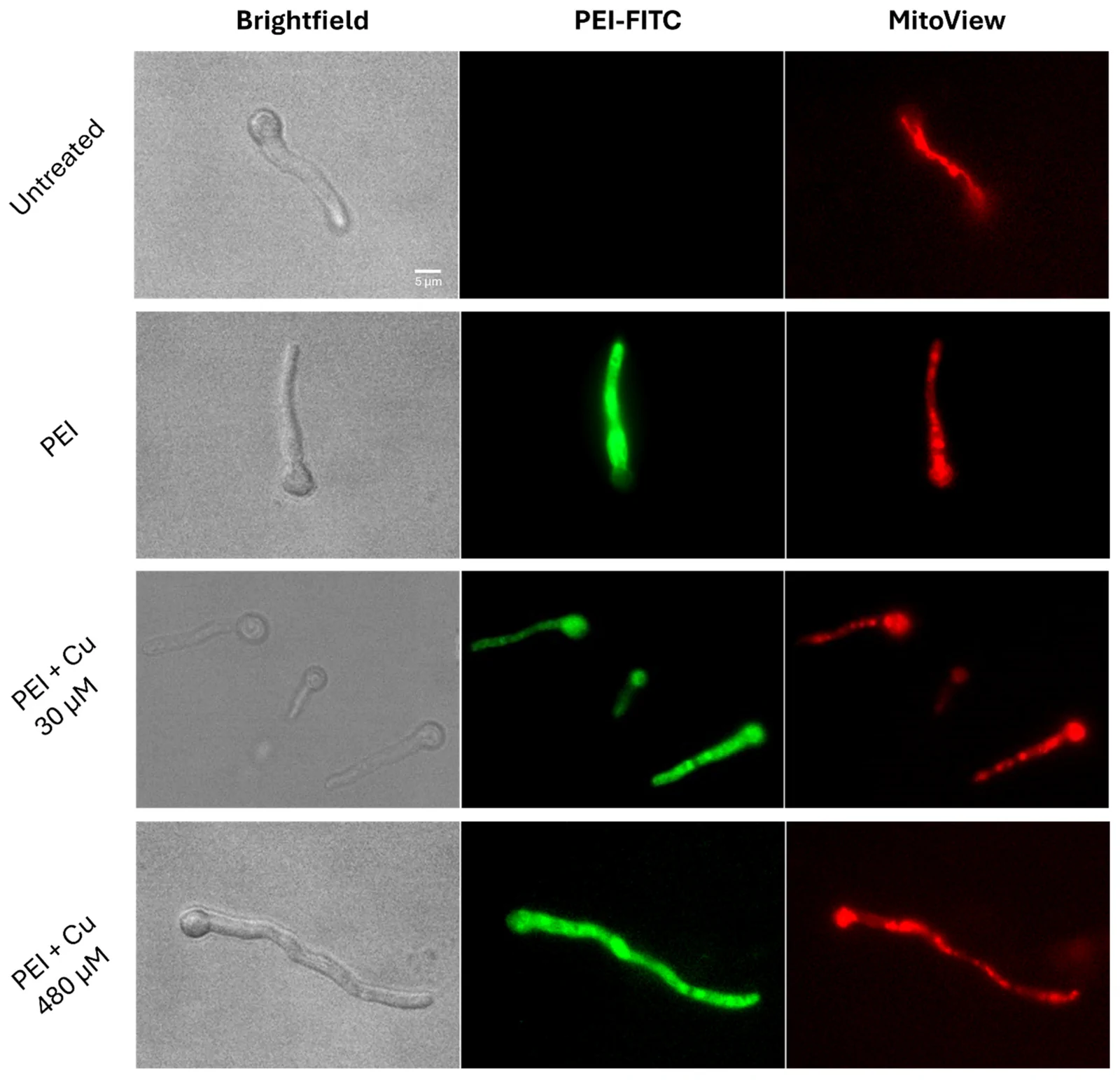
Cu^2+^ protection against PEI toxicity depends on the developmental stage of the cells. Representative epifluorescence microscopy images of 14 h *A. nidulans* germlings untreated or pre-incubated with Cu^2+^ (30 or 480 µM, 3 h, 25 °C) and subsequently treated with PEI-FITC (5 µg mL^−1^) for 1–2 h, and stained with MitoView 650. Untreated germlings (no PEI-FITC, top row) serve as controls for baseline FITC signal and healthy (filamentous) mitochondrial morphology. Cu^2+^ concentrations of 30 µM and 480 µM are shown as representative of the full range tested (30–480 µM); intermediate concentrations (80, 160 and 320 µM) produced comparable results. At all concentrations tested, Cu^2+^ pre-treatment did not alter the pattern of PEI-FITC accumulation or mitochondrial network fragmentation relative to non-pre-treated controls, indicating that pre-acquired intracellular Cu^2+^ does not confer protection against PEI in actively growing germlings regardless of the Cu^2+^concentration. The highest non-cytotoxic Cu^2+^ concentration tested was 480 µM, while the basal level of Cu^2+^ in growth media used (MM) was 1.6 μM. Scale bar at 5 μm.

Having established that Cu^2+^ pre-treatment is sufficient for its protective effect on quiescent conidia (Section 3.1, Figure 1B), we explored whether such a protection extends to actively growing germlings. Germlings pre-incubated with various Cu^2+^ concentrations (30–480 µM, 3 h, 25 °C) and subsequently exposed to PEI-FITC showed no reduction in PEI-FITC accumulation and no preservation of mitochondrial network integrity relative to non-pre-treated controls, across all Cu^2+^ concentrations tested (Figure 6). Therefore, Cu^2+^ pre-treatment protection appears to be developmental stage-dependent: Cu^2+^ pre-treatment was effective in quiescent conidia but not in actively growing germlings, indicating that the metabolic and/or structural state of the cell is critical for the efficacy of intracellular Cu^2+^-mediated protection against PEI toxicity.

## 4. Discussion

In the present study, we investigated how exogenous Cu^2+^ modulates PEI antifungal activity against *A. nidulans* and highlight PEI-dependent early membrane-associated events. Our results demonstrate that exogenous Cu^2+^ protects *A. nidulans* conidia and growing germlings from PEI-induced loss of viability in a concentration-dependent manner and that this protection conferred before or during the earliest stages of PEI-membrane interaction is independent of its extracellular or intracellular administration. The narrow time window of Cu^2+^ protective action, the developmental stage- dependent protection by pre-acquired Cu^2+^, the eisosome-independent rescue of the phenotype, and the convergent evidence from FM4-64 kinetics and ergosterol redistribution experiments, together suggest that the fungal plasma membrane is the primary target of PEI, and Cu^2+^ acts mainly during the early stages of PEI action, preceding vacuolar alterations and mitochondrial fragmentation. Taken together with our previous findings [21,22], the aforementioned observations support a model in which PEI initially perturbs the plasma membrane integrity and ergosterol organization. This disruption triggers downstream cellular responses including ROS production and mitochondrial fragmentation eventually leading to cell death.

Our findings are consistent with previous studies showing that PEI can exert antimicrobial effects through disruption of microbial membranes. In bacterial systems, PEI has been associated with membrane depolarization and increased membrane permeability, while studies in *Candida albicans* have demonstrated membrane damage and depolarization following PEI exposure [20,34,35]. However, the present findings extend these observations to a filamentous fungus and provide evidence for additional alterations in fungal membrane organization, including redistribution of ergosterol and a distinct accumulation at the hyphal tip. Thus, although membrane perturbation appears to be a common feature of PEI activity across diverse microbes, the specific cellular consequences may depend on the architecture and physiology of the target microorganism.

Rescue of *A. nidulans* conidia from PEI-induced death by their co-treatment with exogenous Cu^2+^ in a concentration-dependent manner, with a near-complete protection at 160 µM, extends our previous findings using PEI-Cu complexes [22]. We now provide evidence that free, non-chelated Cu^2+^ also modulates PEI antifungal activity, independently of prior PEI-Cu coordination. Importantly, similar susceptibility to PEI of conidia maintained in Cu^2+^-depleted medium (MM − Cu) to those grown in standard MM in the absence of supplemental Cu^2+^ indicates that the physiological Cu^2+^ concentration present in standard MM (1.6 µM) is insufficient to significantly hinder PEI toxicity, and that Cu^2+^-mediated protection requires concentrations above physiological levels. The dose–response relationship observed (no significant rescue at 30 µM; partial at 60 µM; near-complete at 160 µM) indicates the existence of a minimal concentration required for effective protection. The protective effect of copper against antifungal compounds has been established: previous studies in *Candida albicans* have likewise shown that Cu^2+^ availability modulates antifungal susceptibility and the cellular response to antifungal stress [36,37,38]. Interestingly, the direction of this interaction differs from that reported for fluconazole in *C. albicans*, where modest changes in copper availability can potentiate antifungal activity and substantially alter the cellular transcriptional response to drug stress [36,37,38]. This contrast suggests that the effect of Cu^2+^ on antifungal susceptibility is not necessarily universal, but may depend on both the antifungal agent and the cellular processes targeted by it. In the case of the non-azole, polymer PEI, our results indicate that elevated Cu^2+^ instead attenuates early membrane-associated toxicity.

Full protection observed by pre-loading conidia with excess Cu^2+^, followed by bulk removal of extracellular Cu^2+^ before PEI exposure suggests that an intracellular Cu^2+^ pool, rather than extracellular Cu^2+^ per se, is sufficient for the protective effect. This argues against a model in which Cu^2+^ acts solely by forming extracellular PEI-Cu complexes that reduce the effective concentration of membrane-active polymer in the medium, and points instead to an intracellular Cu^2+^-dependent cellular state that reduces susceptibility to PEI. To this point is important to note that although the washout experiments demonstrate a persistent concentration-dependent protective phenotype, they do not definitively rule out the presence of residual Cu^2+^ ions bound to the cells. A fraction of copper ions may remain attached despite standard washing steps and subsequently interact with or neutralize PEI during the exposure phase. Therefore, although the downstream phenotypic assays performed are consistent with the clear presence of a cellular factor in the observed protection from PEI, local physicochemical neutralization of PEI remains a possible additional factor that cannot be ruled out without analytical determination (e.g., ICP-MS). Furthermore, whether Cu^2+^-dependent preconditioning involves specific alterations in oxidative stress responses, plasma membrane composition, or broader cellular stress adaptation remains unclear. Given the complex interplay between copper homeostasis and iron assimilation pathways in filamentous fungi, further genetic and biochemical studies are necessary to fully resolve the precise intracellular metallostatic mechanisms involved.

The fungal cell actively regulates intracellular Cu^2+^ levels through high-affinity importers (CtrA, CtrC in *A. nidulans*), chaperones and the efflux pump CrpA [25,26,27,28,29,30,31,32,33,34,35,36,37,38,39]. Their involvement in the loss of fungal viability induced by PEI will provide further insight into the mechanism underlying PEI activity.

The filipin III staining experiments provide a structural correlation of PEI’s membrane-disruptive activity at the level of lipid organization. In untreated *A. nidulans* germlings, filipin fluorescence was mainly confined to the PM, consistent with the established PM-predominant localization of ergosterol in filamentous fungi [40]. PEI treatment caused redistribution of filipin signal from the PM to intracellular vesicular/punctate compartments, qualitatively similar to the redistribution observed with the antifungal AmB. Given that AmB exerts its antifungal activity by directly binding and sequestering membrane ergosterol, leading to channel-mediated membrane permeabilization [9], the parallel filipin redistribution phenotype following PEI treatment suggests that PEI likewise perturbs ergosterol organization at the PM. Our findings do not establish whether this redistribution reflects a direct effect on ergosterol organization or a secondary consequence of PEI-induced membrane damage. Ergosterol redistribution from the PM has been shown to have functional effects on membrane integrity and pathogenicity: disruption of ergosterol spatial organization in *Cryptococcus neoformans* through deletion of the sterol transfer protein Ysp2 causes PM and cell wall malformations and reduces virulence [41]. Cu^2+^ presence prevents PEI-induced filipin redistribution, which further supports the finding that Cu^2+^ acts during the early membrane-associated stages of PEI toxicity.

A distinct feature of PEI-treated cells, not observed in untreated controls or AmB-treated cells, was the polarized enrichment of filipin signal at the hyphal tip. The hyphal tip is a known site of ergosterol enrichment in filamentous fungi, where sterol-rich membrane microdomains support the polarized vesicular trafficking and exocytosis required for apical growth [40,42,43]. The spatial accordance between this filipin enrichment pattern and the preferential accumulation of PEI-FITC at the hyphal tip, suggests that PEI preferentially associates with the apical region of actively growing germlings. This targeting is mechanistically distinct from AmB, which binds ergosterol across the membrane irrespective of growth polarity.

The evidence that PEI interferes with PM organization is in agreement with the FM4-64 internalization assay which provided a kinetic view of the earliest effects of PEI on plasma membrane lipid internalization and the stage at which Cu^2+^ protection occurs. PEI accelerated FM4-64 internalization, with the majority of germlings displaying late endosomal/vacuolar dye distribution within 2.5 min of dye loading, while FM4-64 progressively labels endosomal compartments over 15–20 min, in the absence of PEI [33], consistent with our previous results that PEI rapidly compromises PM integrity [21,22]. In contrast, the presence of Cu^2+^ restored FM4-64 localization kinetics to those of untreated cells, whereas delayed Cu^2+^ addition failed to do so. Importantly, this membrane protection was independent of eisosome assembly, as full Cu^2+^-mediated rescue was retained in the eisosome-lacking *pilAΔ* strain, despite previous observations that elevated Cu^2+^ induces eisosome formation in *A. nidulans* [Sophianopoulou and Athanasopoulos, unpublished observations], indicating that Cu^2+^-induced eisosome formation and Cu^2+^-mediated protection represent distinct cellular responses.

The consistent reduction in PEI-FITC fluorescence following Cu^2+^ treatment should be interpreted with caution, as Cu^2+^ directly quenched FITC fluorescence in a cell-free control. Nevertheless, the protection conferred by Cu^2+^ pre-treatment, in which extracellular Cu^2+^ was removed before PEI administration, supports an actual protective effect rather than an artifact due to fluorescence quenching or extracellular PEI-Cu complex formation alone and an independent contribution of intracellular Cu^2+^.

Protection of quiescent conidia but not actively growing germlings by Cu^2+^ pre-treatment against PEI toxicity reveals a developmental stage-dependence. In growing cells, the same Cu^2+^ pre-treatment partially restored normal FM4-64 localization following 10 min of PEI exposure but failed to prevent PEI-FITC accumulation and mitochondrial fragmentation following prolonged PEI treatment (1–2 h), suggesting that Cu pre-treatment could transiently protect early membrane-associated events but did not provide sustained protection during prolonged PEI exposure. This difference is in accordance with the depletion or redistribution of the pre-acquired intracellular Cu^2+^ pool in actively growing germlings (characterized by high rates of polarized vesicular trafficking, active membrane synthesis at the hyphal tip, and continuous endocytic cycling [33,34,35,36,37,38,39,40,41,42,43,44]). In quiescent conidia, by contrast, the absence of active growth and membrane activity may allow a pre-acquired Cu^2+^ pool to remain effective throughout the 4 h PEI exposure used in CFU assays. Taken together with our prior characterization of PEI-Cu chelate complexes [22], the present results provide additional insights on how Cu^2+^ intersects with the antifungal mechanism of PEI. In PEI-Cu chelates, Cu^2+^ pre-coordinated to PEI delayed but did not abolish antifungal activity, coinciding with delayed PEI internalization and a reduced rate of growth inhibition [22]. The present study extends these findings by demonstrating that free exogenous Cu^2+^, above certain concentrations, also modulates PEI activity through the fungal cell, rather than exclusively through direct interaction with PEI. This conclusion is supported by the finding that intracellular Cu^2+^ remained protective after bulk removal of extracellular Cu^2+^. Second, this cellular protection is temporally and developmentally constrained. Together, our studies support that Cu^2+^ modulates PEI activity by extracellular coordination to PEI, which delays its antifungal activity, and intracellular Cu^2+^-dependent protection of the fungal cell. These findings reconcile the apparently different effects of chelated and free Cu^2+^ and suggest a framework for understanding how Cu^2+^ modulates the antifungal activity of PEI. Exogenously administered high concentration of copper significantly reduces the activity of PEI as an antifungal agent. As copper levels are known to increase by host immune cells during a fungal infection [45,46], in a potential cutaneous application of PEI, the simultaneous administration of a chelating-agent that removes copper from the point of infection will allow PEI to act more efficiently. Since a small amount of copper protects human dermal fibroblasts from PEI [22], administration should be strictly regulated.

## 5. Conclusions

In conclusion, we have demonstrated that exogenous Cu^2+^ protects *A. nidulans* from PEI toxicity through a concentration-dependent, timing-restricted, developmentally regulated and eisosome compartmentalization-independent mechanism that operates primarily through early plasma membrane integrity perturbations, accompanied by alterations in ergosterol organization and consequently mitochondrial fragmentation and cell death [22]. In combination with our previous findings on PEI and PEI-Cu complexes, the present study advances understanding of the mechanism of PEI antifungal activity and reveals how Cu^2+^ reduces this activity. Together, these findings provide a mechanistic framework for the rational design of improved PEI-based antifungal strategies.

## Data Availability

The original contributions presented in this study are included in the article. Further inquiries can be directed to the corresponding author.

## Supplementary Materials

The following supporting information can be downloaded at: https://www.mdpi.com/article/10.3390/jof12100746/s1, Figure S1: Representative chemical structure of hyperbranched polyethyleneimine (PEI); Table S1: Current state of knowledge and outstanding questions regarding the antimicrobial activity of PEI and the role of Cu^2+^ in modulating antifungal activity.
