# Supplementary material for: Copper Availability Modulates Early Membrane-Associated Events in Polyethyleneimine Antifungal Activity in Aspergillus nidulans
Source: J Fungi (Basel). 2026 Oct 4;12(10):746. doi: 10.3390/jof12100746 (PMC13705712; doi:10.3390/jof12100746)
Supplement: Supplementary file 1 [file jof-12-00746-s001.zip › jof-4577921-supplementary.pdf]

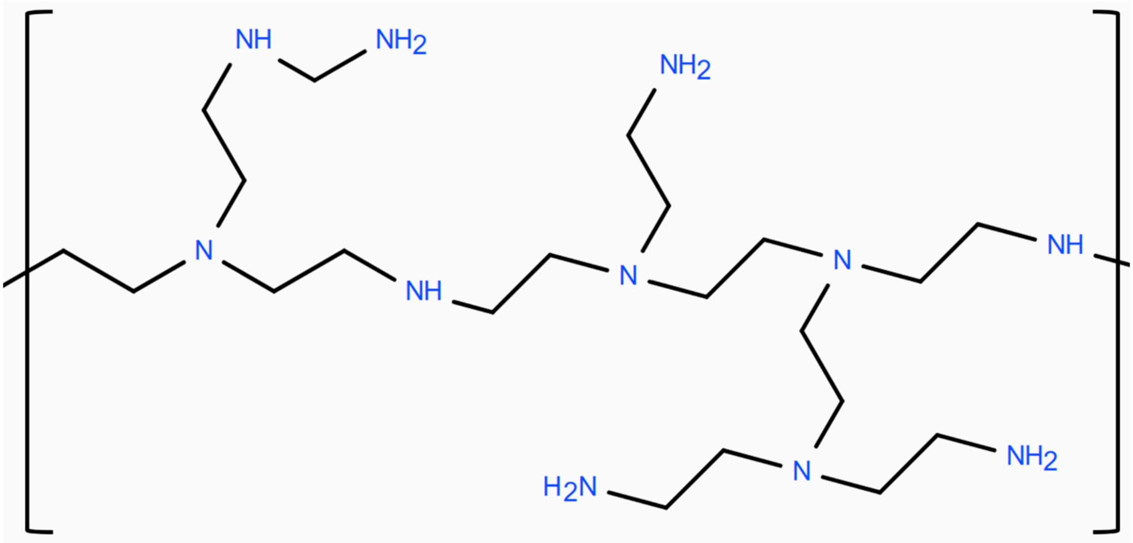

Figure S1: Representative chemical structure of hyperbranched polyethyleneimine (PEI). The structure illustrates the hyperbranched architecture of PEI and the presence of primary, secondary, and tertiary amine groups. The PEI used in this study was hyperbranched PEI (Lupasol® WF, 25 kDa, 99%, CAS 9002-98-6).

**Established knowledge**	**Evidence**	**What remains unknown**
PEI is an antimicrobial polymer	PEI is a potent antimicrobial polymer. In *Escherichia coli*, PEI has been shown to permeabilize the bacterial outer membrane, increasing membrane permeability and sensitizing cells to hydrophobic antibiotics [20].	Whether similar membrane-associated events account for PEI toxicity in fungi
PEI is antifungal against filamentous fungi	Our previous studies demonstrated that hyperbranched PEI is strongly antifungal against both quiescent conidia and germlings of *A. nidulans* [21,22].	The specific cellular and membrane-associated mechanism underlying PEI antifungal activity
Cu^{2+} can modulate antifungal responses	Copper availability can alter fungal susceptibility and cellular responses to antifungal drugs. In *Candida albicans*, changes in Cu^{2+} availability modulate fluconazole tolerance, and this effect does not require direct Cu–drug complex formation [36, 37].	Whether Cu^{2+} availability similarly modulates the activity of a membrane-active polymer such as PEI
PEI–Cu^{2+} complexes remain antifungal	Preformed PEI–Cu^{2+} complexes retain antifungal activity against *A. nidulans*. They reduce the hyphal growth rate in *A.*	Whether Cu^{2+} itself modulates PEI activity independently of pre-

	nidulans germlings compared to PEI alone [22].	existing PEI–Cu^{2+} complex formation

Table S1: Current state of knowledge and outstanding questions regarding the antimicrobial activity of PEI and the role of Cu^{2+} in modulating antifungal activity. The table summarizes established findings from previous studies and highlights the specific knowledge gaps addressed by the present study.
